# Heart Metastases of Clear Cell Renal Cell Carcinoma

**DOI:** 10.3390/diagnostics13091600

**Published:** 2023-04-30

**Authors:** Katarzyna Romejko, Adam Rytel, Tomasz Rozmyslowicz, Stanisław Niemczyk

**Affiliations:** 1 Department of Internal Diseases, Nephrology and Dialysis, Military Institute of Medicine-National Research Institute, 128 Szaserów Street, 04-141 Warsaw, Poland; arytel2@wim.mil.pl (A.R.); sniemczyk@wim.mil.pl (S.N.); 2 Department of Pathology and Laboratory Medicine, Perelman School of Medicine, University of Pennsylvania, R.217 John Morgan Building, 3620 Hamilton Walk, Philadelphia, PA 19104, USA; rozmyslo@pennmedicine.upenn.edu

**Keywords:** clear cell renal cell carcinoma, metastases, heart

## Abstract

Renal cell carcinoma (RCC) is a common genitourinary cancer. Of the several histologic types of RCC, clear cell renal cell carcinoma (ccRCC) is the most frequent. Due to the development of imaging methods such as computed tomography (CT) or magnetic resonance imaging (MRI), the incidence of ccRCC diagnosis has increased rapidly. However, up to one third of patients at prime diagnosis of ccRCC are at metastatic stadium of the disease. Metastases of ccRCC are found mostly in the lungs, bones and liver. Metastasis of ccRCC to the heart is an uncommon clinical situation. We present a rare case of metastatic stadium of ccRCC with metastases to heart tissue visualized in transthoracic echocardiography.

Renal cell carcinoma (RCC) is a common type of kidney cancer, diagnosed in over 400,000 individuals worldwide each year, accounting for 2–3% of all cancers [1,2]. RCC is more frequent in men, and the average age at diagnosis is 64 [3]. Among the American population, Africans, Hispanics and Native Americans have a higher risk of RCC compared to White Americans [4]. Modifiable risk factors for RCC include smoking, obesity, poorly-controlled hypertension, a diet rich with meat-based protein and fat, alcohol, occupational exposure and certain drugs [5,6,7]. Clear cell renal cell carcinoma (ccRCC) is the most common subtype, accounting for 70% of cases, and originates from the proximal tubule traits [8,9]. Patients with ccRCC may remain asymptomatic, and more than 50% of cases are diagnosed incidentally [10]. The classic triad of RCC symptoms, including flank pain, macrohematuria and palpable abdominal mass in physical examination, is seen in only 10% of patients, while 20% present paraneoplastic symptoms such as hypercalcemia, policythemia, hypertension or Cushing’s syndrome [11]. Treatment options for ccRCC include radical or partial nephrectomy and ablative methods, but up to 30% have metastases at the time of diagnosis [12]. Metastases of ccRCC are commonly found in the abdominal lymph nodes, lungs, liver and bones and are resistant to chemiotherapy and radiotherapy [13]. However, new therapeutic options, such as tyrosine kinase inhibitors and monoclonal antibodies targeting vascular endothelial growth factor, have shown promise in treating advanced ccRCC [14]. Immunotherapy is also a promising strategy, but results vary [15]. Despite new ways of treatment, the 5-year survival rate for metastatic ccRCC remains low at approximately 10%, with a slight recent increase from 7 to 12% [16,17].

We present a case report of a man with the metastatic stage of ccRCC and with metastases to the heart.

A 58-year-old man with end-stage renal disease, treated with hemodialysis, after right-sided nephrectomy due to ccRCC, with metastases of ccRCC to the lungs, stomach and left adrenal gland, who was disqualified from receiving oncological treatment was admitted to the hospital due to acute deterioration. On admission, the patient reported weakness, dizziness and daily episodes of bradycardia, with a heart rate of 40 to 44 beats per minute. However, on physical examination, the patient had a regular heart rate of 70 beats per minute, elevated blood pressure of 180/100 mmHg and normal lung fields on auscultation. 

Laboratory tests showed an elevated concentration of the C-reactive protein (9.5 mg/dL; reference range, 0.0–0.8 mg/dL) as well as increased levels of creatinine (5.3 mg/dL; reference range, 0.7–1.2 mg/dL) and urea (97 mg/dL; reference range, 18–55 mg/dL, respectively). White blood cell count was normal (5.7 × 10^9^/L; reference range, 4.3–9.64 × 10^9^/L), but red blood cell count was decreased (2.58 × 10^12^/L; reference range, 4.36–5.78 × 10^12^/L), with a reduced hemoglobin concentration (7.5 g/dL, reference range, 13.5–17 g/dL). An electrocardiogram revealed a Wenckebach type of second-degree atrioventricular block, which after a few hours progressed to third-degree atrioventricular block with a heart rate of 35 beats per minute. A transthoracic echocardiography examination was performed, which revealed changes in heart tissue diagnosed as metastases of ccRCC to the heart. The largest metastatic lesion, with dimensions of 47 mm × 18 mm, was visualized in the left ventricle and was attached to the interventricular septum (Figure 1A,B). The metastasis infiltrated the mitral valve, did not cause its stenosis, but led to severe mitral regurgitation (Figure 1C). The metastasis was also connected with the non-coronary cusp of the aortic valve, causing stenosis with a maximum velocity through the valve of 3 m/s and a mean pressure gradient of 22 mmHg. Echocardiography also showed that the lesion measured 34 mm × 8 mm attached to the largest metastatic mass, which was mobile and balloted through the aortic valve (Figure 1D). Additionally, two metastases measuring 10 mm × 8 mm and 8 mm × 7 mm were visualized at the top of the largest metastatic mass, and both of these were round and mobile (Figure 1E). 

The patient was cardiologically consulted and, based on the echocardiography examination, the metastases of ccRCC to the heart were diagnosed. Due to anemia, a blood transfusion was performed. Because of the increase of C-reactive protein concentration (11.6 mg/dL; reference range, 0.0–0.08 mg/dL), symptoms of pneumonia and pulmonary inflammatory changes in the chest X-ray, antibiotic therapy was implemented, resulting in a reduction in inflammatory markers and improvement in clinical status. Due to the third-degree atrioventricular block, the patient underwent implantation of a Single Chamber Ventricular (VVI) pacemaker, which was performed without complications. The patient was discharged from the hospital after stabilization.

As the patient was disqualified from oncological treatment, palliative therapy was continued. The patient visited the dialysis ward three times a week, and hemodialysis procedures were performed. Due to anemia, he received blood transfusions. The patient died six months and one week after the diagnosis of ccRCC cardiac metastases.

Cardiac metastases are more frequent than primary cardiac tumors, and they primarily originate from lung, breast and hematologic malignancies [18,19]. Cardiac metastases from ccRCC are rare. In metastatic ccRCC, the tumor can extend to renal vein and inferior vena cava and in this way even to heart. We decided to show our case of ccRCC metastases reaching the heart because of its educational value. Clinical manifestations of cardiac metastases are due to their locations, but heart tumors may also be asymptomatic and can be diagnosed post-mortem. The clinical presentation in this case was arrhythmia such as a Wenckebach type of second-degree atrioventricular block, which progressed to a third-degree atrioventricular block. A transthoracic echocardiography visualized numerous lesions in heart tissue, and some of them were mobile. Despite the metastatic stage of the disease and possibility of complications caused by the presence of multiple metastases in heart, the cardiology team decided to implant a Single Chamber Ventricular pacemaker to prevent life-threatening arrhythmias and to prolong the patient’s life, which was successfully performed. We did not decide to make a biopsy of the visualized lesions because of the possibility of complications due to invasive diagnostic procedures. The results of diagnosis would not change the management of the patient and would not provide operative or non-operative treatment options. The echocardiographic findings also indicated that the observed changes in the heart tissue were not consistent with infective endocarditis.

## Figures and Tables

**Figure 1:**
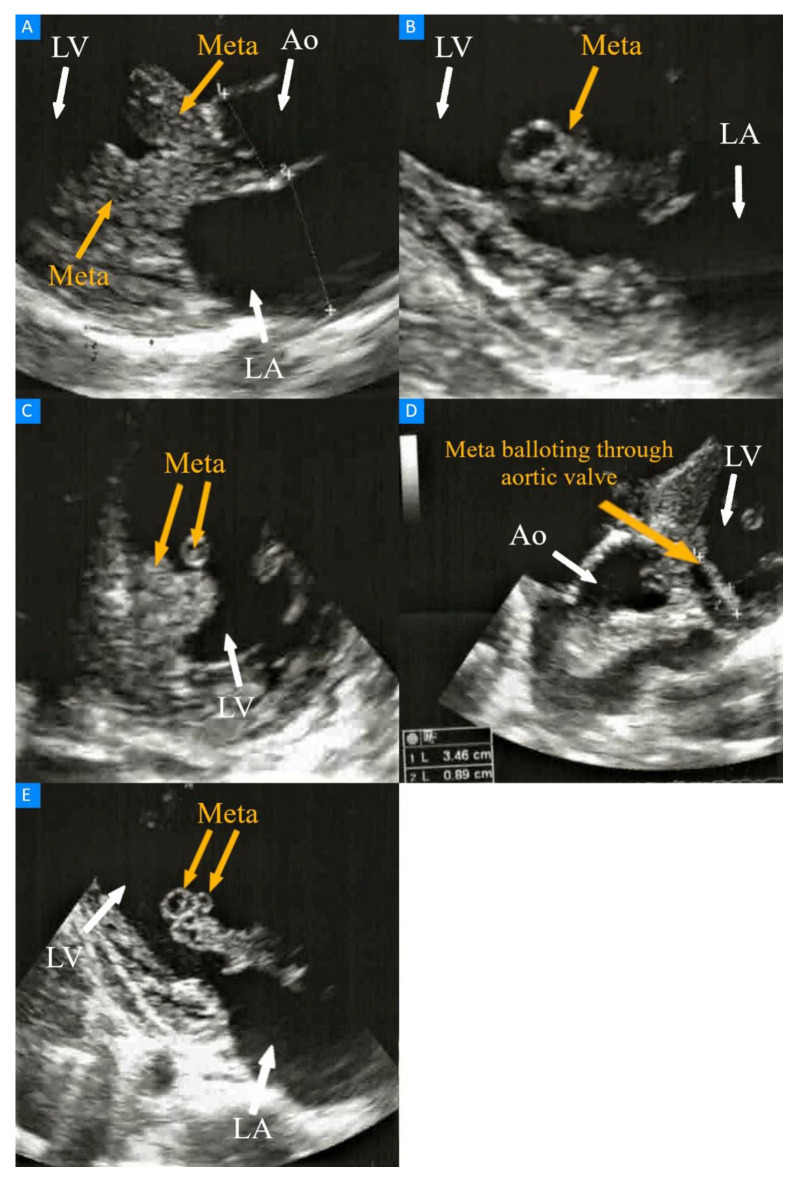
Transthoracic echocardiography visualizing metastases of ccRCC to the heart. (**A**) Modified parasternal long-axis view, metastasis in the left ventricle infiltrating interventricular septum; 1—the dimension of the aortic root, 2—the dimension of the left atrium. (**B**) Modified parasternal long axis view, metastasis in the left ventricle. (**C**) Parasternal short axis view, metastasis infiltrating the mitral valve. (**D**) Modified apical 5 chamber view visualizing the mobile metastatic lesion of 35 mm × 8 mm which balloted through the aortic valve. (**E**) Modified apical 3 chamber view visualizing two round and mobile metastases.

## Data Availability

The data presented in this study are available on request from the corresponding author. The data are not publicly available due to Polish General Data Protection Regulation.

## Abbreviations

Meta: metastasis; LV, left ventricle; LA, left atrium; Ao, aorta.
